# Nanotechnology in Plant Science: To Make a Long Story Short

**DOI:** 10.3389/fbioe.2019.00120

**Published:** 2019-05-29

**Authors:** Ilaria Sanzari, Antonietta Leone, Alfredo Ambrosone

**Affiliations:** ^1^Faculty of Engineering and the Environment, University of Southampton, Southampton, United Kingdom; ^2^Department of Pharmacy, University of Salerno, Fisciano, Italy

**Keywords:** nanomaterials, nanogels, plant nanobiotechnology, plant protection, nanosensors, advanced genetic engineering

## Abstract

This mini-review aims at gaining knowledge on basic aspects of plant nanotechnology. While in recent years the enormous progress of nanotechnology in biomedical sciences has revolutionized therapeutic and diagnostic approaches, the comprehension of nanoparticle-plant interactions, including uptake, mobilization and accumulation, is still in its infancy. Deeper studies are needed to establish the impact of nanomaterials (NMs) on plant growth and agro-ecosystems and to develop smart nanotechnology applications in crop improvement. Herein we provide a short overview of NMs employed in plant science and concisely describe key NM-plant interactions in terms of uptake, mobilization mechanisms, and biological effects. The major current applications in plants are reviewed also discussing the potential use of polymeric soft NMs which may open new and safer opportunities for smart delivery of biomolecules and for new strategies in plant genetic engineering, with the final aim to enhance plant defense and/or stimulate plant growth and development and, ultimately, crop production. Finally, we envisage that multidisciplinary collaborative approaches will be central to fill the knowledge gap in plant nanotechnology and push toward the use of NMs in agriculture and, more in general, in plant science research.

## Introduction

Nanomaterials have unique physicochemical properties and provide versatile scaffolds for functionalization with biomolecules. Moreover, certain NMs such as gold and magnetic nanoparticles as well as polymeric or hybrid NMs have shown to respond to external stimuli achieving a spatiotemporal controlled release of macromolecules. For these reasons, over the last two decades, engineered nanomaterials have been successfully tested and applied in medicine and pharmacology, especially for diagnostic or therapeutic purposes (Bruchez et al., 1998; Tang et al., 2006; Perrault et al., 2009). More recently, the field of nanotechnology is gaining an increased interest in plant science, especially for the application of nanomaterials (NMs) as vehicles of agrochemicals or biomolecules in plants, and the great potential to enhance crop productivity (Khan et al., 2017).

It is reasonable to argue that the potentiality and the benefits of the application of NMs in plant sciences and agriculture are still not fully exploited, due to some bottlenecks, which can be briefly summarized as follows: (i) the need to design and synthesis safe NMs which do not interfere negatively with plant growth and development (Sabo-Attwood et al., 2012); (ii) the lack of knowledge on the exact mechanisms of NMs uptake and mobilization in plants (Ranjan et al., 2017) and, (iii) the lack of multidisciplinary approaches, necessary for the design and the implementation of nanotechnology applications in plants.

## Nanomaterials in Plant Science

According to ASTM standards, Nanomaterials (NMs) can be defined as natural or manufactured materials, typically ranging between 1 and100 nm (Astm E2456 - 06, 2012). NMs have a small size and a high surface-to-volume ratio, which confer to them remarkable chemical and physical properties in comparison to their bulk counterparts (Roduner, 2006). NMs have unique and versatile physicochemical properties, which makes their use suitable in different fields, such as life science, electronics and chemical engineering (Jeevanandam et al., 2018). Recently, nanotechnology is gaining interest also in plant science, due to the need to develop miniaturized efficient systems to improve seed germination, growth and plant protection to abiotic and biotic stresses (Wang et al., 2016).

**Figure 1:**
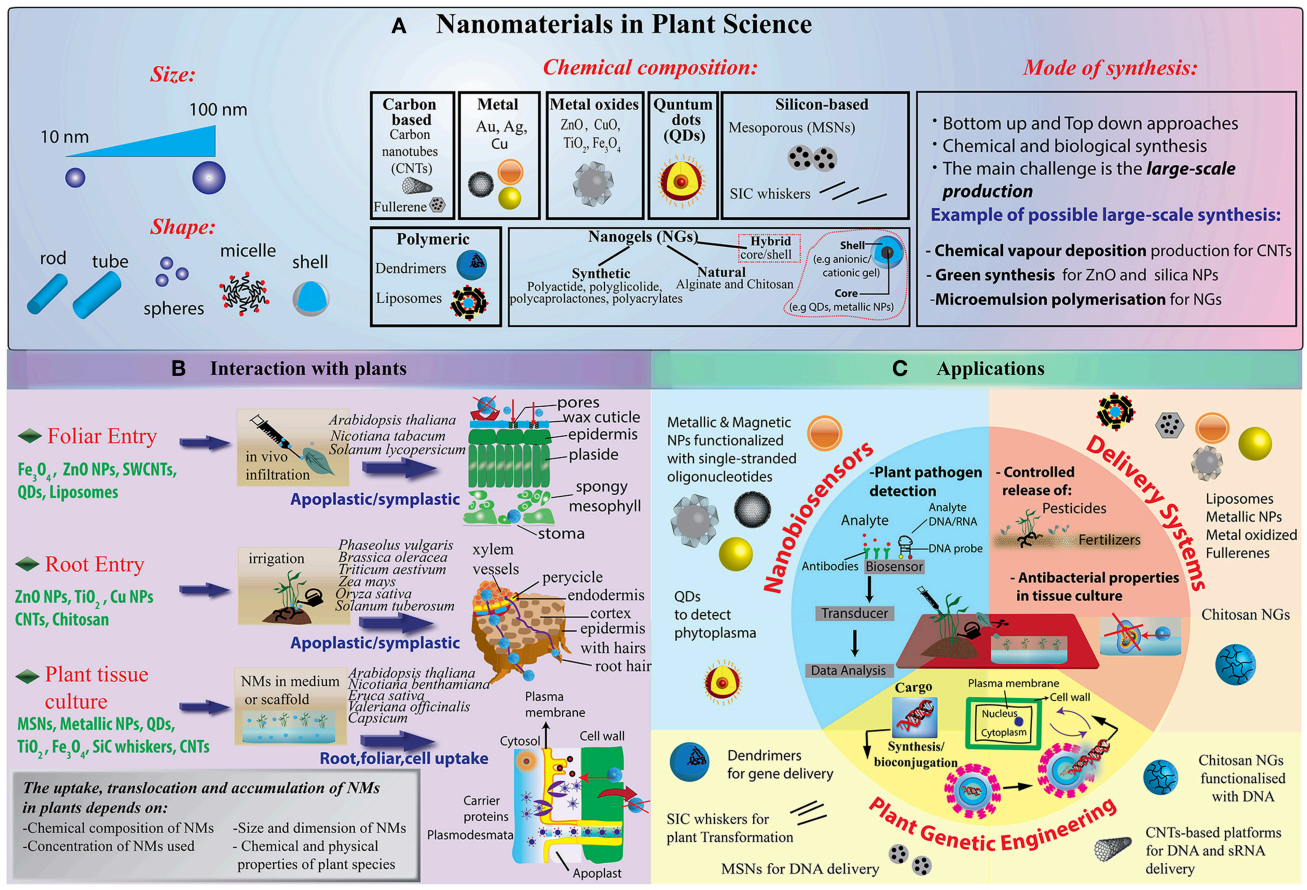
**(A)** Illustration of NMs grouped into several categories: carbon-based NMs such as fullerenes and carbon nanotubes, including single-walled carbon nanotubes (SWCNTs) or multi-walled carbon nanotubes (MWCNTs); metallic NPs, including metals such as gold (Au), silver (Ag), aluminum (Al); metal oxides (ZnO, CuO, TiO_2_, Fe_2_O3, SiO_2_, etc.); quantum dots (QDs); dendrimers, which are three dimensional polymer network immensely branched with low polydispersity and liposomes and nanogels. With the development of new techniques for chemical synthesis, it is possible to synthesize NMs not only with a symmetrical (spherical) shape but also having a variety of different nanoforms, such as nanoclays (polypropylene nanoclay systems) and nanoemulsions (lipophilic nanoemulsions), tubes, rods, disks, bars, and sheets. **(B)** Schematization of different NP delivery methods and translocation in plants. Nanoparticle can be administered both at foliar and root system. Once penetrated the external layers, they move through the symplastic or apoplastic routes and reach different organs and tissues. **(C)** Currently, the main focus of the publications in plant science deals with the use of NPs as biosensors or biomolecules nanocarriers for crop production and protection under controlled conditions. New advances in DNA/miRNA/siRNA delivery have found limited application in plant so far, while new nanotechnology tools addressing technical concerns in genome editing strategies are strongly demanded.

Metallic nanoparticles (NPs), such as gold (Au), and silver (Ag) NPs, have been widely introduced in plant science for different applications (Figure 1A). Their chemical synthesis is quite costly and requires the use of hazardous chemicals (Viswanath and Kim, 2015; Rastogi et al., 2019). However, greener approaches based on the use of plant extract as well as ionizing radiation chemistry in aqueous solutions have been developed (Abedini et al., 2013). Also oxidized NMs, such as MgO, CaO, ZnO, and TiO_2_ NMs, have been widely proposed, thanks to their superior electrical, catalytic and light absorption properties (Jahan et al., 2018). Over the recent years, the interest in polymeric nanomaterials is predominantly increasing due to their biocompatibility, low-cost synthesis and capability to response to external stimuli (Baskar et al., 2018). Core/Shell NPs are also available and can be manufactured with a variety of combination of materials such as inorganic/inorganic, inorganic/organic, organic/inorganic, and organic/organic materials. The choice of the shell of the NPs strongly depends on the end application and use (Ghosh Chaudhuri and Paria, 2012). For example, polymeric shells have been proposed to improve the biocompatibility of the NPs (Nath et al., 2008). NPs with a nanostructured shell have been also synthesized, such as mesoporous silica nanoparticles (NPs) made from a mesoporous structure with a highly functionalizable surface area (Torney et al., 2007).

Nanogels (NGs) are a new category of NM with a growing interest in the nanotechnology community. They have excellent physicochemical properties, colloidal stability, high encapsulation capacity of biomolecules (bioconjugation), and stimuli-responsiveness (pH, temperature, etc.). NGs are defined as nano-sized ionic and non-ionic hydrogels made of synthetic or natural polymeric chains, chemically or physically cross-linked (Molina et al., 2015; Neamtu et al., 2017). NGs possess a high water content (70–90% of the entire structure), a high degree of porosity and high load capacity. The most common NGs are chitosan, alginate, poly(vinyl alcohol), poly(ethylene oxide), poly(ethyleneimine), poly (vinylpyrrolidone), poly(N-isopropylacrylamide). NGs with hybrid structures, made of polymeric or non-polymeric materials can be obtained (Molina et al., 2015). Hybrid NGs have been classified in: (i) nanomaterial– nanogel, which are synthesized by incorporation of nanosized materials such as magnetic or carbonaceous nanoparticles, and (ii) polymer–nanogel composites, which include interpenetrated networks (IPNs), copolymer, and core-shell particles (Molina et al., 2015). The main advantage of IPNs and copolymer NGs relies on their stimuli-responsiveness, whereas core-shell NGs are more promising for encapsulating biomolecules and drug delivery.

## Nanoparticle Uptake, Translocation, and Biological Impact in Plants

Applications of nanotechnology strategies in plants need a preventive accurate evaluation of nanoparticle-plant interactions, including the comprehension of the mechanisms of their uptake, translocation and accumulation, together with the assessment of potential adverse effects on plant growth and development. Plant uptake of NPs is hardly predictable, depending on multiple factors related to the nanoparticle itself (size, chemical composition, net charge and surface functionalization), but also on the application routes, the interactions with environmental components (soil texture, water availability, microbiota), the constraint due to the presence of a cell wall, the physiology and the multifaceted anatomy of individual plant species. Most of the previous studies in plants deal with the uptake of small metal and metal oxide NPs, due to the wide use in industry and to the easy detection and tracking by microscopy techniques (González-Melendi et al., 2008). However, compared to the great wealth of information available in metazoans, only a handful of integrated comparative analyses have been conceived to establish the contribution of the physicochemical features (e.g., size, charge, coatings, etc.) of NPs in plant-nanoparticle interaction (Zhu et al., 2012; Song et al., 2013; Moon et al., 2016; Vidyalakshmi et al., 2017; García-Gómez et al., 2018).

### Delivery Methods and Primary Interactions at the Plant Surface

Basically, engineered nanomaterials can be applied either to the roots or to the vegetative part of plants, preferentially to the leaves (Figure 1B). At the shooting surface, NPs can be taken up passively through natural plant openings with nano- or microscale exclusion size, such as stomata, hydathodes, stigma and bark texture (Eichert et al., 2008; Kurepa et al., 2010). However, additional plant anatomical and physiological aspects need to be considered to better understand the dynamics of NP-plant interactions. For instance, shoot surfaces are generally covered by a cuticle made of biopolymers (e.g., cutin, cutan) and associated waxes, which function as a lipophilic barrier to protect above-ground plant primary organs, leaving access only through natural openings (Figure 1B). Dynamics of NPs at the cuticle level are poorly investigated, but at present, this barrier appears to be an almost impenetrable layer to nanoparticles, although nano-TiO2 has been shown to be able to produce holes in the cuticle (Larue et al., 2014; Schwab et al., 2016). Trichomes on plant organs can affect dynamics at the plant surface by entrapping NP on the plant surface and thus increasing the permanence time of exogenous materials on tissues. Damages and wounds may also function as viable routes for NP internalization in plants in both aerial and hypogeal parts (Al-Salim et al., 2011). Delivery methods also seem to influence NP uptake efficiency in plants. As recently reported, the aerosol application promotes higher internalization rates of different nanoparticles with respect to NP drop cast in watermelon (Raliya et al., 2016). Also, leaf lamina infiltration strategies may force NM penetration in plant tissues as reported for single-walled carbon nanotubes (Giraldo et al., 2014) and resulted to be functional for gene delivery (Demirer et al., 2018). At the root level, rhizodermis lateral root junctions may provide easy access to NMs, especially near the root tip, while upper parts are impermeable due to the presence of suberin (Chichiriccò and Poma, 2015). Generally, the dynamics of NP uptake appear to be more complex in the soil compared to the plant aerial part. Several factors, as the presence of mucilage and exudates, symbiotic organisms, and soil organic matter may influence NPs availability. For instance, root mucilage and exudates normally excreted into the rhizosphere play a dual role: they may promote NP adhesion to the root surface, which in turn may enhance NP internalization rate or, conversely, these gel-like substances may also trigger NP trapping and aggregation (Avellan et al., 2017; Milewska-Hendel et al., 2017). Recent observations, by means of X-ray computed nanotomography and enhanced dark-field microscopy combined with hyperspectral imaging, have demonstrated that root border cells and associated mucilage tend to trap gold NPs irrespective of particle charge, while negatively charged NPs are not sequestered by the mucilage of *Arabidopsis thaliana* root cap and translocate directly into the root tissue (Avellan et al., 2017).

The presence of symbiotic bacteria and fungi in the soil have been demonstrated to play controversial roles as well; in general, they enhance accumulation of different types of heavy metal NPs in true grasses, but reduce nano-Ag and nano-FeO uptake in legumes (Whiteside et al., 2009; Feng et al., 2013; Guo and Chi, 2014).

### Nanoparticle Mobilization in Plant

Once penetrated the plant outer protective layers and regardless of aerial or hypogeal exposure, NMs have two mobilization routes in the plant: apoplastic and symplastic paths (Figure 1B). Apoplastic transport occurs outside the plasma membrane through the cell wall and extracellular spaces, whereas symplastic movements involve the transport of water and solutes between the cytoplasm of adjacent cells connected by plasmodesmata and sieve plate pores.

Apoplastic transport has been demonstrated to promote radial movement of NMs, which may move NPs to the root central cylinder and the vascular tissues, and promoting their movement upwards the aerial part (González-Melendi et al., 2008; Larue et al., 2014; Sun et al., 2014; Zhao et al., 2017). This manner of NP translocation is instrumental for applications requiring systemic NP delivery. However, the Casparian strip, a longitudinally oriented layer made of lignin-like structures, prevent the completion of this radial movement in the root endodermis (Sun et al., 2014; Lv et al., 2015). To bypass this natural barrier, water and another solute switch from apoplastic to the simplastic path. Similar abilities to circumvent the block at Casparian strip have been documented for different kinds of NPs as reviewed in Schwab et al. (2016). This may happen especially in those anatomical regions where the Casparian strip is not yet properly formed, such as root tips and root lateral junctions (Lv et al., 2019).

The symplastic transport of NPs requires that at some point NPs penetrate inside the cells. The presence of a rigid plant cell wall creates a physical barrier to the cell entry and makes the intracellular delivery of NPs in plants much more difficult with respect to animal cells. Basically, the cell wall is a multi-layered framework of primarily cellulose/hemicellulose microfibrils and scaffold proteins, creating a porous milieu which acts as a narrow selective filter with a mean diameter <10 nm, with some exception up to 20 nm (Carpita et al., 1979). Actually, this is a critical point and currently represents one of the main hurdles to the design and the implementation of bioengineering tools in plants (Cunningham et al., 2018). However, different types of nanoparticles with a mean diameter between 3 and 50 nm and carbon nanotubes have been demonstrated to easily pass through the cell wall in many plant species (Liu et al., 2009; Kurepa et al., 2010; Chang et al., 2013; Etxeberria et al., 2019).

Subsequent cell internalization may occur preferentially by endocytosis (Valletta et al., 2014; Palocci et al., 2017), although alternative cell entry mechanisms, such as those based on pore formation, membrane translocation or carrier proteins already described in cells (Nel et al., 2009; Lin et al., 2010; Wang et al., 2012) and in invertebrate models (Marchesano et al., 2013) need to be further elucidated in plant cells. For instance, it has been demonstrated that Multi-Walled Carbon nanotubes (MWCNTs) may enter in *Catharanthus roseus* protoplasts by an endosome-escaping uptake mode (Serag et al., 2011).

Once in the cytoplasm, cell to cell movements of NPs are facilitated by plasmodesmata, membrane-lined cytoplasmic bridges with a flexible diameter (20–50 nm), which ensure membrane and cytoplasmic continuity among cells throughout plant tissues. Transport of NPs with variable sizes through plasmodesmata has been described in Arabidopsis, rice, and poplar plant species (Lin et al., 2009; Geisler-Lee et al., 2013; Zhai et al., 2014).

Through the symplastic and apoplastic pathways, small particles can reach the xylem and phloem vessels and translocate in the whole plant to different tissues and organs. Remarkably, organs like flowers, fruits and seeds normally have a strong capability to import fluids from the phloem (sink activity) and tend to accumulate NMs. Besides plant toxicity, NP accumulation in specialized organs raises another important issue related to their safe use in human and animal consumption (Pérez-de-Luque, 2017).

Worth mentioning from an application perspective, studies in different crops, such as maize, spinach, cabbage, reported the ability of metal-NPs to penetrate seeds and translocate into the seedlings, without significant effects on seed viability, germination rate, and shoot development. These data suggest the possible use of functional NPs for seed priming and plant growth stimulation, also in limiting environmental conditions (Zheng et al., 2005; Rǎcuciu and Creangǎ, 2009; Pokhrel and Dubey, 2013).

### Nanoparticle Phytotoxicity

The comprehension of NM toxicity in crop plants is still at dawn, but it is crucial for the implementation of innovative agro-nanotech tools and products (Servin and White, 2016). Current NP studies in plants have investigated unrealistic scenarios, such as short-term and high dose exposure, often in model media and plant species, gathering contradictory results (Miralles et al., 2012). Basically, most of the studies have demonstrated that in cultivated species (e.g., tomato, wheat, onion, and zucchini) excess of metal-based NPs trigger an oxidative burst by interfering with the electron transport chain as well as by impairing the reactive oxygen species (ROS) detoxifying machinery, with genotoxic implications (Dimkpa et al., 2013; Faisal et al., 2013; Pakrashi et al., 2014; Pagano et al., 2016). As a consequence, plant secondary metabolism, hormonal balance and growth are often negatively affected. Interestingly, recent transcriptome analyses revealed that exposures to different types of NPs (e.g., zinc oxide, fullerene soot, or titanium dioxide) exposure represses a significant number of genes involved in phosphate-starvation, pathogen and stress responses, with possible negative effects on plant root development and defense mechanisms in *A. thaliana*. A recent systems biology approach, including omics data from tobacco, rice, rocket salad, wheat, and kidney beans, confirmed that metal NMs provoke a generalized stress response, with the prevalence of oxidative stress components (Ruotolo et al., 2018). These data suggest that further studies based on high-throughput analysis of genetic and metabolic responses, triggered by NP exposure, are necessary to shed light on many aspects of NP phytotoxicity in crops, even in absence of overt toxicity at the phenotypic level (Majumdar et al., 2015). In light of these evidence, it appears fair to exploit for future applications in plants engineered NMs for which a safe profile has been already established in animal systems, such as soft polymeric NPs.

## Current Applications in Plant Science

As mentioned above, while nanotechnology innovation is running fast in many fields of life science, smart applications in plant and agricultural science still lag behind (Wang et al., 2016). In this section, we review the most significant current approaches (schematized in Figure 1C), in particular, those inherent to biosensing, delivery of agrochemicals and genetic engineering. Representative applications for different types of NPs are also listed in Table 1 together with a brief description of their positive effects and drawbacks in plant species.

**Table 1:** Major applications of different nanomaterials in plant and respective positive/negative impact.

**NMs and relative size (nm)**	**Plant species**	**Beneficial impact**	**Negative impact (if any)**	**Application**	**References**
**Carbon-Based**					
Fullerene 1–2 nm	*Cucurbita pepo* *Glycine max* *Momordica charantia* *Populus deltoids* *Solanum lycopersicum* *Zea mays*	Increase biomass and fruit yield in bitter melon Reducing the accumulation of pesticides in tomato, corn, soybean and zucchini	At high concentrations, reduced corn and soybean biomass Increased uptake of trichloroethylene in poplar	Drug delivery in agriculture	Kole et al., 2013 Ma and Wang, 2010 De La Torre-Roche et al., 2013
MWCNTs SWCNTs 4–30 nm	*Arabidopsis thaliana* *Eruca sativa* *Gossypium hirsutum* *Nicotiana bentahmiana* *Nicotiana tabacum* *Oryza sativa* *Solanum lycopersicum*	Enhanced germination, growth and flowering of tomato Enhanced tobacco callus growth and metabolic production High efficiency in DNA delivery in tobacco, arugula, cotton	Chromatin condensation and apoptosis in Arabidopsis and rice protoplasts Stress-inducing ROS accumulation in protoplasts	Plan genetic engineering	Shen et al., 2010 Ivanov et al., 2016 Khodakovskaya et al., 2013 Demirer et al., 2018 Serag et al., 2013
**Metallic NPs**					
Ag 1–40 nm	*Brassica juncea* *Citrullus lanatus* *Cucurbita pepo* *Oryza sativa* *Raphanus sativum* *Zea mays*	Stimulation of seedling growth in watermelon and zucchini Stimulation of shoot and root length, and improvement of photosynthic quantum efficiency in brown mustard	Toxic effects on corn root growth Oxidative stress and reduction of seedling growth in radish	Study of Plant-NP interactions	Almutairi and Alharbi, 2015 Sharma et al., 2012
Au 5–20 nm	*Arabidopsis thaliana* *Gloriosa superba* *Hordeum vulgare* *Oryza sativa* *Solanum lycopersicum*	No toxicity in barley and tomato Improved seed germination and vegetative growth in climbing lily	Strong Accumulation of nanoparticles in root	Study of Plant-NP interactions Imaging	Milewska-Hendel et al., 2017 Dan et al., 2015 Avellan et al., 2017 Gopinath et al., 2014 Zhu et al., 2012
Cu 10–30 nm	*Cucumis sativus* *Phaseolus radiatus* *Triticum aestivum* *Sorghum bicolor*		Decrease in the total biomass, High NP accumulation and gene expression deregulation in cucumber root Extensive bioaccumulation and toxicity in mung bean, wheat and sorghum	Study of Plant-NP interactions	Alawadhi et al., 2018 Lee et al., 2008
**Metal-Based NPs**					
CdSe/ZnS QDs 1–10 nm	*Allium cepa L* *Arabidopsis thaliana* *M. sativa*	Efficient pathogen detection when used as biosensors	Enhanced ROS production NP transfer into trophic chain Reduced plant cell viability Decreasing of root length	Study of Plant-NP interactions Imaging/Fluorescent detection Nanobiosensors	Santos et al., 2010 Modlitbová et al., 2018 Koo et al., 2015 Rad et al., 2012
CuO 20–30 nm	*Arabidopsis thaliana* *Cucumis sativus* *Oryza sativa* *Triticum aestivum*	Essential nutrients in plant growth due to the presence of Cu	Phytotoxicity with increasing number of ROS enzymes Dose dependent reduction in shoot and root	Plant Genetic Engineering	Alawadhi et al., 2018 Shi et al., 2013 Wang et al., 2016
Fe_3_O_4_ <100 nm	*Triticum aestivum* *Zea Mays*	Positive effects on plant height and leaf area wheat	Brown spots on leaves at higher volume fractions in corn	Nanofertilizers Study of Plant-NP interactions	Fathi et al., 2017 Racuciu, 2012
TiO_2_ <5 nm	*Arabidopsis thaliana* *Oryza sativa* *Spinacia oleracea*	Increases plant growth in spinach by improving nitrogen metabolism in spinach Promote seed germination		Nanofertilizers	Gao et al., 2008 Liu et al., 2019 Kurepa et al., 2010
ZnO 30–200 nm	*Allium cepa* *Arabidopsis thaliana* *Brassica napus* *Cucumis sativus* *Lactuca sativa* *Lolium perenne* *Oryza sativa* *Raphanus sativus* *Zea mays*	Reduction of flowering time and improvement of seed production in onion Increase plant growth Beneficial effect on seed germination (in low dose)	Modifications in microbial enzymatic ac- Tivities in soil At high concentrations, inhibition of root growth in radish, rape, ryegrass, lettuce, corn and cucumber	Nanopesticides Micronutrients delivery	Laware and Raskar, 2014 Lin and Xing, 2007
**Silicon Based**					
MSNs 2–50 nm	*Allium cepa* *Nicotiana tabacum* *Zea mays*	Controlled release of chemicals and nucleic acids		Plant Genetic Engineering Delivery of pesticides and fertilizers	Torney et al., 2007 Valenstein et al., 2013 Rastogi et al., 2019
SiC whiskers 50–60 nm	*Gossypium hirsutum*	Efficient genetic transformation		Plant genetic engineering	Asad and Arsh, 2012
**Polymeric Nanomaterials**					
Chitosan-based NPs 90 nm	*Camellia sinensis* *Triticum aestivum*	Biodegradable and biocompatible materials Antimicrobial activity Plant growth stimulation		Delivery of nanofertilizers and herbicides Plant genetic engineering	Malerba and Cerana, 2018 Islam et al., 2017 Abdel-Aziz et al., 2016 Dasgupta et al., 2015
Dendrimers 10–20 nm	*Agrostis* *stolonifera*	Endosomal escape of delivered DNA		Plant Genetic Engineering	Pasupathy et al., 2008 Kretzmann et al., 2017
Liposomes 100 nm	*Nicotiana benthamiana* *Solanum lycopersicum*	Enhanced delivery of encapsulated DNA by membrane fusion Protection of nucleic acids from nuclease activity Cell specific targeting	DNA/liposome complexes, high toxicity, poor stability, and rapid clearance	Delivery of nutrients and DNA	Karny et al., 2018

### Biosensors

NMs have been applied to develop biosensors or they have been used as “sensing materials” in the fields of crop biotechnology, agriculture, and food industry (Duhan et al., 2017; Chaudhry et al., 2018). Different categories of nanosensor types have been tested in plants, including plasmonic nanosensors, fluorescence resonance energy transfer (FRET)-based nanosensors, carbon-based electrochemical nanosensors, nanowire nanosensors and antibody nanosensors. Although the use of nanosensors in plants is at an initial stage (Rai et al., 2012), interesting reports have proposed the use of NMs as tools for detection and quantification of plant metabolic flux, residual of pesticides in food and bacteria, viral and fungal pathogens. Recently, it has been reported the fabrication of a fluorometric optical onion membrane-based sensor for detection of sucrose based on the synthesis of invertase-nanogold clusters embedded in plant membranes (Bagal-Kestwal et al., 2015). In addition, single-walled carbon nanotubes (SWNTs) have been exploited for near-infrared fluorescence monitoring of nitric oxide in *A. thaliana* (Giraldo et al., 2014). FRET probes conjugated to polystyrene NPs have been also designed to quantify and recognize the phytoalexins (Dumbrepatil et al., 2010).

As above mentioned, NMs-based biosensors are very promising as they allow rapid detection and precise quantification of fungi, bacteria and viruses in plants (Duhan et al., 2017). For example, fluorescent silica NPs combined with antibody was designed for diagnosing *Xanthomonas axonopodis pv. vesicatoria*, which causes bacterial spot disease in Solanaceae plants (Yao et al., 2009). Recently, Au NPs have been proposed from Lau et al. as DNA biochemical labels to detect *Pseudomonas syringae* in *A. thaliana* by differential pulse voltammetry (DPV) on disposable screen-printed carbon electrodes (Lau et al., 2017). Similarly, fluorescently labeled-DNA oligonucleotide conjugated to Au NPs were employed in the diagnosis of the phytoplasma associated with the flavescence dorée disease of grapevine (Firrao et al., 2005). Finally, smart nanosensors are also available for mycotoxin detection; for instance, the 4mycosensor is a competitive antibody-based assay successfully introduced in the market to test the presence of ZEA, T-2/HT-2, DON, and FB1/FB2 mycotoxin residues in corn, wheat, oat and barley (Lattanzio and Nivarlet, 2017).

### Controlled Release of Agrochemicals and Nutrients

NMs can be applied to the soil as nanostructured fertilizers (nanofertilizers, as for Fe, Mn, Zn, Cu, Mo NPs) or can be used as enhanced delivery systems to improve the uptake and the performance of conventional fertilizers (nutrients and phosphates) (Liu and Lal, 2015). Even though nanofertilizers and NM-enhanced fertilizers are very promising for agriculture, the use of nanotechnology in fertilizer supply is very scanty (DeRosa et al., 2010).

Hydroxyapatite nanoparticles, used as phosphorous nanofertilizers, enhance the soybean growth rate and seed yield by 33 and 20%, compared to a regular P fertilizer (Liu and Lal, 2015). In addition, nanofertilizers can be released at slower rates which may contribute to maintain the soil fertility by reducing the transport of these nutrients into a runoff or ground water and decreasing the risks of environmental pollution and toxic effects due to their over-application (Liu and Lal, 2015).

Metallic nanoparticles based on Iron oxide, ZnO, TiO_2_, and copper have been directly applied as nanofertilizers in soil by irrigation or via foliar applications in different plants, such as mung bean plant, cucumber and rape (Gao et al., 2006; Tarafdar et al., 2014; Saharan et al., 2016; Verma et al., 2018). Similarly, MWNTs used as soil supplements increased twice the number of flowers and fruits in tomato plants likely through the activation of genes/proteins essential for plant growth and development (Khodakovskaya et al., 2013). Despite these intriguing evidence, the use of nanofertilizers is still debatable. Accumulation in treated soils may pose a threat to soil microbial communities such as small invertebrates, bacteria and fungi (Frenk et al., 2013; Waalewijn-Kool et al., 2013; Shen et al., 2015; Simonin et al., 2016; Goncalves et al., 2017). This impact on the agro-ecosystem reasonably discourages the use of metallic nanoparticles in agriculture.

Only recently, a natural polymer, such as chitosan NPs, have been used for controlled release of nitrogen, phosphorus and potassium in wheat by foliar uptake (Abdel-Aziz et al., 2016). The use of organic NPs is more acceptable in terms of environmental pollution. However, their effective advantages for nutrient supply over traditional fertilization methods need more robust evidence (Liu and Lal, 2015).

On the other hand, pesticides delivered by nanomaterials generally have increased stability and solubility and enable slow release and effective targeted delivery in pest management (Duhan et al., 2017). Organic and polymeric NPs in the form of nanospheres or nanocapsules have been used as nanocarriers for herbicide distribution (Tanaka et al., 2012). In particular, polymeric NPs, such as Poly(epsilon-caprolactone), present good properties of biocompatibility and have been repeatedly used for the encapsulation of atrazine herbicide (Tanaka et al., 2012). In another study, chitosan nanoparticles loaded with three triazine herbicides have shown reduced environmental impact and low genotoxic effects in *Allium cepa* (Grillo et al., 2015).

### Nanomaterials for Plant Genetic Engineering

As stated above, the cell wall represents a barrier to the delivery of exogenous biomolecules in plant cells. To overcome this barrier and achieve plant genetic transformation, different strategies based on *Agrobacterium* transformation or biolistic methods are worldwide used for DNA delivery in plant cells. Limitations to these approaches rely on narrow host range and plant extensive damages, which often inhibit plant development.

Most of the pioneering studies for nanomaterial-based plant genetic engineering have been conducted in plant cell cultures. For example, Silicon Carbide-Mediated Transformation has been reported as a successful approach to deliver DNA in different calli (tobacco, maize, rice, soybean and cotton) (Armstrong and Green, 1985; Wang et al., 1995; Serik et al., 1996; Asad and Arsh, 2012; Lau et al., 2017).

Although lagged behind the advancements achieved in animal systems, results reported recently in plants are proving that NMs may overcome the barrier of the cell wall in adult plants and reduce the drawbacks associated with current transgene delivery systems.

One seminal study proved that dsRNA of different plant viruses can be loaded on non-toxic, degradable, layered double hydroxide (LDH) clay nanosheets or BioClay. The dsRNAs and/or their RNA breakdown products provide protection against the Cauliflower Mosaic Virus (CMV) in sprayed tobacco leaves, but they also confer systemic protection to newly emerged, unsprayed leaves on viral challenge 20 days after a single spray treatment in tobacco (Mitter et al., 2017). More in general, this is a proof of concept for species-independent and passive delivery of genetic material, without transgene integration, into plant cells for different biotechnology applications in plants.

A successful stable genetic transformation has been achieved in cotton plants via magnetic nanoparticles (MNPs). β-glucuronidase (GUS) reporter gene- MNP complex were infiltrated into cotton pollen grains by magnetic force, without compromising pollen viability. Through pollination with magnetofected pollen, cotton transgenic plants were successfully generated and exogenous DNA was successfully integrated into the genome, effectively expressed, and stably inherited in the offspring obtained by selfing (Zhao et al., 2017).

In another recent paper, carbon nanotubes scaffolds applied to external plant tissue by infusion were used to deliver linear and plasmid DNA, as well as siRNA, in *Nicotiana benthamiana, Eruca sativa, Triticum aestivum*, and *Gossypium hirsutum* leaves and in *E. sativa* protoplasts, resulting in a strong transient Green Fluorescent Protein (GFP) expression. Moreover, the same authors reported that small interfering RNA (siRNA) was delivered to *N*. *benthamiana* plants constitutively expressing GFP, causing a 95% silencing of this gene (Demirer et al., 2018).

The first and promising approach of genome editing mediated by mesoporous silica nanoparticles (MSNs) has been recently proposed. MSNs have used as carriers to deliver Cre recombinase in *Zea mays* immature embryos, carrying *loxP* sites integrated into chromosomal DNA. After the biolistic introduction of engineered MSNs in plant tissues, the *loxP* was correctly recombined establishing a successful genome editing (Valenstein et al., 2013).

## Conclusions and Future Perspectives

Herein, we have discussed various facets of using NMs in plant sciences. In the last years, it has been demonstrated that nanotechnology has made huge progress in the synthesis of NMs and their application in medicine for diagnosis and therapy. On the other side, the application of NMs for plants is still poor. Recent outcomes and current applications suggest that more studies are necessary for this direction to optimize the synthesis and biofunctionalization of NMs for plant applications, but also to elucidate deeper the mechanisms of plant uptake and improving the sustainability for agro-ecosystems and human health. Interestingly, applications need to be extended to address uncovered important aspects of plant physiology. For instance, nanobiosensors for detecting secondary metabolites or phytoregulators in real time may provide advances in monitoring plant development and interactions with the environment, especially in limiting growth conditions.

Despite the huge progress in plant genetics, the delivery of exogenous DNA and/or enzymes for genome editing remain a big challenge. New strategies based on nanoparticle-mediated clustered regularly interspersed palindromic repeats—CRISPR associated proteins (CRISPR-Cas9) technology, as those tested in other biological systems (Lee et al., 2017; Glass et al., 2018), would provide ground-breaking innovation in plant genetics.

On the base of consolidated evidence reported in cell and animal models, soft materials, like nanogels, and polymeric nanostructures should be further exploited as favorable candidates to develop new strategies for controlled release of biomolecules and plant genome editing. Owing to their safe profile, high loading capacity and excellent cargo protection from degradation polymeric and hydrogel-based NPs have shown undeniable advantages in drug delivery. Moreover, this kind of NMs has been elegantly employed to achieve a controlled (spatial and temporal) release of cargos triggered by external stimuli (e.g., UV, NIR, acoustic waves etc.) (Ma et al., 2013; Ambrosone et al., 2016; Linsley and Wu, 2017) in cell and animal models. These outstanding results suggest that the huge potential of soft nanomaterials remains almost unexplored in plants. Besides a few successful attempts for agrochemicals delivery above-mentioned and listed in Table 1, more efforts are needed to design strategies and smart tools based on polymeric or hybrid materials for applications in plants. Of course, a careful analysis of manufacturing scalability and cost-effectiveness needs to be undertaken before the extensive use of polymeric nanomaterials in agriculture.

As a final remark, the delay in plant nanotechnology might be overcome by encouraging the activation of multidisciplinary approaches for the design and the synthesis of smart nanomaterials. To this aim, joint collaborative initiatives, merging complementary professional competencies such those of plant biologists, geneticists, chemists, biochemists, and engineers, may disclose new horizons in phytonanotechnology.

## Author Contributions

IS and AA conceived the idea and organized this mini review. All authors wrote the manuscript and approved the contents for publication.

### Conflict of Interest Statement

The authors declare that the research was conducted in the absence of any commercial or financial relationships that could be construed as a potential conflict of interest.
